# The second United Nations high-level meeting on the fight to end TB: action is needed to turn the tide by 2030

**DOI:** 10.5588/pha.23.0042

**Published:** 2023-09-21

**Authors:** L. Ditiu, G. N. Kazi

**Affiliations:** 1 Stop TB Partnership, Geneva, Switzerland;; 2 International Union Against Tuberculosis and Lung Disease, Paris, France

TB affects billions of lives either directly or indirectly, damages national economies and continues to overwhelm health systems: it is unacceptable that TB has been allowed to persist in modern times. World leaders will gather to focus on TB at this year’s United Nations General Assembly at a pivotal moment – if we fail to accelerate the anti-TB response, our chances of eliminating this terrible affliction will be greatly diminished. Following the 2018 High-Level Meeting on the Fight Against Tuberculosis, the political commitments endorsed by the UN General Assembly were ambitious and manifold.^1^ Articulated within an official UN political declaration were commitments to successfully treat 40 million people with TB, including 1.5 million people with drug-resistant TB (DR-TB), provide preventive therapy to at least 30 million people, mobilise US$13 billion a year for TB care, and invest at least US$2 billion a year in research. These were to be delivered by 2022, but then COVID-19 struck. Historic levels of resources were mobilised to support the COVID-19 response, including the use of public health infrastructure previously used for TB care. At the same time, policies that restricted social movement, combined with people’s concerns about exposure to COVID-19 reduced access to health centres, disrupting the delivery of TB diagnosis, treatment and care.^2^,^3^ According to the WHO, the number of people diagnosed and treated for TB plummeted, with a 15% reduction in people treated for drug-resistant TB (DR-TB), a 21% decrease in people receiving TB preventive treatment and a 9% decrease in TB spending over the previous year.^4^ In 2021, WHO called attention to the first reported increase in global TB mortality in more than a decade – news that reverberated around the world.^5^ Adding to these challenges are new armed conflicts, a global migration crisis, increasing urbanisation and a spike in global poverty—all of which complicate the TB response.

At this year’s General Assembly, global leaders are expected to present their reflections and reaffirm their commitment to ending the TB pandemic. This must lead to action and catalyse interventions that accelerate progress toward TB elimination by 2030, the deadline for reaching the UN Sustainable Development Goals. From our perspective, the following are priorities for action that risk being overlooked in this critical phase of the global TB response:

1. The TB pandemic is multifaceted, requiring support for policies that enable biomedical interventions as well as interventions to address the social, environmental and economic determinants of TB.^3^ TB overwhelmingly affects countries, communities, families and individuals impacted by poverty and marginalisation. To leave no one behind, leaders must support national TB responses that are equitable, inclusive, gender-sensitive, rights-based and people-centred. The COVID-19 response provides an informative case study when it comes to equity and access to new tools. Wealthier nations stockpiled millions of vaccine doses to the detriment of people in low- and middle-income countries. Paradoxically, this action likely left the entire world less safe from the pandemic, and wealthier countries eventually saw massive quantities of these unused vaccines expire.^6^ As part of an increase in financing and support for TB research and development, leaders must advance policies and initiatives to ensure equitable access to new TB vaccines, diagnostics, and drugs and digital technologies, and make concerted efforts to reach vulnerable groups.
2. It is heartening to observe that world leaders will collaboratively address various global health challenges during the UN General Assembly session. These challenges include enhancing pandemic preparedness, strengthening health systems, achieving universal health coverage (UHC), and putting an end to TB. These areas overlap and require coordination at the political level. As countries work to recover from the COVID-19 pandemic, significant new investment is needed to speed up progress toward UHC, made available through efficient resource mobilisation and allocation based on where interventions will deliver the greatest good.^7^ Policies should enable TB care services to be integrated with efforts to attain UHC. One way is to require TB services to be included within essential health service packages that are delivered through primary health care.^8^
3. UN High-Level Meetings have positioned other related health challenges (such as HIV/AIDS, non-communicable diseases and antimicrobial resistance) as significant enough to require the highest level of political initiative, so heads of state and government have become increasingly important as public health leaders.^9^ Using their delegating authority and ability to inspire action through their offices, world leaders must mobilise additional political support for ending TB within their countries. As a corollary, they must embrace and foster a government-wide culture of accountability for results. Community led monitoring, which empowers affected communities with tools to analyse barriers to TB services, human rights violations, TB stigma (and other events and trends) and report challenges, has emerged as an invaluable aid to accountability, particularly in high-burden countries. In Pakistan, the salutary effect of involving parliamentarians and developing multi-sectoral accountability frameworks at the policymaking and service delivery tiers has been documented.^10^,^11^ Other countries have shown progress using similar approaches. For example, Indonesia issued a presidential decree to end TB in the country by 2030, creating a multisectoral coordinating team led by the Coordinating Ministry of Human Development and Cultural Affairs.^12^ In India, the drive to eliminate TB has been spearheaded by the Prime Minister himself, instilling a sense of urgency through the establishment of an accelerated national objective to eradicate TB by 2025. In March 2023, Prime Minister Modi launched several new TB initiatives at the One World TB Summit, including the TB Mukt Panchayat Abhiyan Initiative – a campaign to mobilise TB interventions at the community level.^13^ Domestic funding for India’s national TB programme has increased several fold in recent years, supported by public-facing health promotion campaigns, new initiatives in private sector TB care, provision of nutrition and other needs to people affected by TB at a unprecedented scale with an all-of-society approach, and incentives and enablers to provide more holistic care for vulnerable groups, among other interventions.
4. Along with increased support for TB research and development, leaders must support policies and allocate resources to facilitate the rapid dissemination of research data, findings and actionable insights. Effective knowledge dissemination is essential for designing policies, strategies and programmes to accelerate progress against TB, but also for implementing interventions more effectively. For example, Stop TB Partnership’s TB REACH and the Challenge Facility for Civil Society are key initiatives for funding local partners for the exploration of innovative approaches to TB detection, treatment and best practices for transforming the TB response. With more support and systems for dissemination, insights generated by such initiatives can better serve TB programmes and facilitate replication and scale-up of the most effective approaches.
5. As members of the TB community, we have consistently advocated for the regular convening of United Nations summits and high-level meetings. This concerted effort aims to maintain global public health as a prominent item on the agenda for heads of states and governments. Given how fundamental public health is to our collective peace and survival – and considering the challenges and needs outlined above – we reiterate this call. Sustained effort is critical to closing financing gaps, improving global health governance and achieving the vision of health for all.^14^,^15^

Finally, there is more to leadership than its political and economic dimensions. World leaders have an ethical and moral imperative, which they must embrace to end this pandemic by 2030.^16^ For many, this requires a change in mindsets. Those who fully take on the challenge will be able to impress upon citizens the urgent importance and profound benefit of ending TB, and powerfully positioned to move countries towards that overarching goal, together.
